# Efficacy of stumble recovery assistance in a knee exoskeleton for individuals with simulated mobility impairment: A pilot study

**DOI:** 10.1017/wtc.2023.17

**Published:** 2023-09-06

**Authors:** Maura E. Eveld, Shane T. King, Karl E. Zelik, Michael Goldfarb

**Affiliations:** 1 Department of Mechanical Engineering, Vanderbilt University, TN, USA; 2 Department of Physical Medicine & Rehabilitation, Vanderbilt University, TN, USA; 3 Department of Electrical Engineering, Vanderbilt University, TN, USA; 4 Department of Biomedical Engineering, Vanderbilt University, TN, USA

**Keywords:** stumble recovery, fall, lower-limb exoskeleton

## Abstract

Falls due to stumbles are a major cause of injury for many populations, and as such interventions to reduce fall risk have been a key focus of rehabilitation research. However, dedicated stumble recovery assistance in a powered lower-limb exoskeleton has yet to be explored as a fall mitigation intervention. Thus young, healthy adults ( 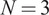 ) were recruited for a stumble recovery experiment to test the efficacy of knee exoskeleton stumble recovery assistance in improving an impaired stumble recovery response (i.e., the elevating strategy response). Leg weights were attached unilaterally to each participant’s shank to simulate walking and stumble recovery impairment, and a unilateral powered knee exoskeleton was worn on the same leg for walking and stumble recovery assistance. Ultimately, knee exoskeleton stumble recovery assistance served to improve participants’ elevating limb kinematics (i.e., increase thigh and knee motion) and reduce overall fall risk (i.e., reduce trunk motion and improve foot placement) during responses relative to their impaired response (i.e., with the leg weights and no assistance), and relative to their response while receiving only walking assistance. This initial exploration provides a first indication that knee exoskeleton stumble recovery assistance is a viable approach to improving an impaired stumble recovery response, which could serve two important use cases: (1) a safety mechanism for existing exoskeleton wearers, who may be less capable of recovering from stumbles due to the added weight or joint impedance of the device; (2) an external stumble recovery aid for fall-prone populations, such as the elderly or stroke survivors.

## 1. Introduction

Falls are a major cause of injury for many populations (Tinetti et al. (1994); Baker and Harvey (1985)). Often falls occur because of a trip or stumble (Berg et al. (1997); Lord et al. (1993); Li et al. (2006); Overstall et al. (1977)). The stumble event, in which the foot unexpectedly encounters an obstacle (in swing phase) that must be cleared to recover, is a common daily-life occurrence that requires a recovery response to avoid a fall. Much rehabilitation research has been devoted to improving stumble recovery and mitigating subsequent injury, particularly for fall-prone populations, such as the elderly (Fuller (2000)), stroke survivors (Jørgensen et al. (2002)), and lower-limb prosthesis users (Miller et al. (2001)).

Several works have characterized stumble recovery and identified the key factors in recovering from a stumble. Ultimately, an individual needs to restore trunk control (arrest forward angular momentum induced by the perturbation) with a sufficient and timely stepping response to recover successfully. Previous works have highlighted the role of both the recovery limb (limb that clears the obstacle) and the support limb (contralateral limb) in recovery success. Pijnappels et al. concluded that the reactive torques of the support limb of healthy adults enable the necessary push-off reaction, thus reducing the forward angular momentum of the body and providing more time for the elevating step (Pijnappels et al., 2005b, 2005c). However, a study with older adults with less adequate push-off responses (Pijnappels et al. (2005a)) found that the participants compensated with better positioning of the recovery (elevating) limb (i.e., longer stride). They proposed that improving the forward swing of the recovery limb, which also works to counter induced forward angular momentum, could be a target for fall prevention training. Grabiner et al. (1993) also highlighted the role of the recovery limb. Knee flexion reduces the moment of inertia of the leg, which increases thigh flexion velocity; thigh flexion and knee extension velocities are integral in providing a sufficient stepping response to recover. Thus, an intervention at the knee joint of the recovery limb (flexion/extension assistance) has the potential to improve recovery responses.

So far, interventions regarding stumble recovery have primarily involved training protocols. Muscle strength training has been proposed as a means to improve both the support limb’s ability to provide counteracting torques and the recovery limb’s ability to provide sufficient obstacle clearance (Pijnappels et al. (2008); Pavol et al. (2002)). Similarly, exercise and balance-focused training (e.g., Tai Chi (Li et al. (2005))) has been proposed as a means to enhance postural stability, lower-limb strength, and flexibility to improve responses to stumble perturbations. Finally, task-specific perturbation training (via sessions involving repeated treadmill acceleration perturbations) has been shown to not only reduce falls and improve stumble recovery during a laboratory-induced trip for various populations (Grabiner et al. (2012); Pater et al. (2016)) but also mitigate real-world falls (Rosenblatt et al. (2013)). In these works, the authors attribute a learned/trained reduction of trunk motion (flexion and flexion velocity) and increased step length to the improved responses. While these works provide promising and effective interventions, and also identify improvable recovery metrics, these extensive, repeated training protocols may not be feasible (i.e., considering cost, time, ability) for many individuals; furthermore, the long-term effects of such trainings have yet to be determined.

Wearable assistive technology has the potential to improve responses to stumbles without repeated training sessions by providing immediate assistance to the recovery and/or support limb. In fact, such behavior may be important not only as a potential fall prevention intervention for fall-prone populations but also as a safety feature for any lower-limb exoskeleton that provides walking assistance. However, dedicated exoskeleton assistance during stumbles has been sparingly considered in the field thus far. While some works have explored stumble detection and identification techniques (Lawson et al. (2010); Zhang et al. (2011); Shirota et al. (2014a); Eveld et al. (2021a)), only a few have investigated the effect of exoskeleton assistance on balance recovery. Hip (Monaco et al. (2017)) and ankle (Emmens et al. (2018); Bayón et al. (2022)) exoskeleton assistance has been shown to improve stability against balance loss during slips and reduce the effort to maintain balance during pushes, respectively. One study analyzed recovery motion following trips while healthy users wore a bilateral exoskeleton with hip and knee actuation, but only the conditions of continuing walking assistance or stopping walking assistance were analyzed (Akiyama et al. (2020)) (i.e., no dedicated stumble recovery assistance). Thus no study has considered dedicated stumble recovery assistance in a knee exoskeleton during swing-phase obstacle perturbations (i.e., trip/stumble perturbations). Recall that an intervention at the knee joint of the recovery limb may improve responses via (1) knee flexion assistance, which reduces the moment of inertia of the recovery limb, allowing for thigh flexion and thereby assisting with angular momentum reduction, and (2) knee extension assistance, which aids in the speed and length of the recovery step. Previous preliminary results suggest that knee exoskeleton assistance can be coordinated with the user to improve the recovery limb response (Eveld et al. (2021a)); however, this was only tested on a single healthy individual, so the extent to which it improves an impaired response for multiple participants has not been demonstrated.

Therefore, the objective of this work is to examine the extent to which powered knee exoskeleton stumble recovery assistance (applied unilaterally to the recovery limb) can potentially improve an impaired stumble recovery response. In this work, early swing perturbations that require an elevating strategy (as defined in Eng et al. (1994); Eveld et al. (2021b)) to recover are considered (i.e., the tripped limb is the recovery limb). Before introducing such an intervention to fall-prone populations (e.g., elderly, stroke survivors, post-polio patients), who may be less suited for the physical demand of an exploratory study requiring many stumbles, the authors deemed it important to first test this type of intervention in healthy individuals. Consequently, to perform this study it was necessary to first simulate an impairment in order to approximate the gait and recovery response of a mobility-impaired population. Thus, leg weights were added unilaterally to each participant’s shank (i.e., the recovery limb wearing the exoskeleton) to (i) induce a walking impairment and (ii) require more effort in the stepping response during stumble recovery. Given this simulated impairment, this article examines the extent to which a knee exoskeleton with dedicated stumble recovery assistance might improve stumble recovery responses in individuals with mobility impairment.

## 2. Methods

### 2.1. Interventional device

**Figure 1.**
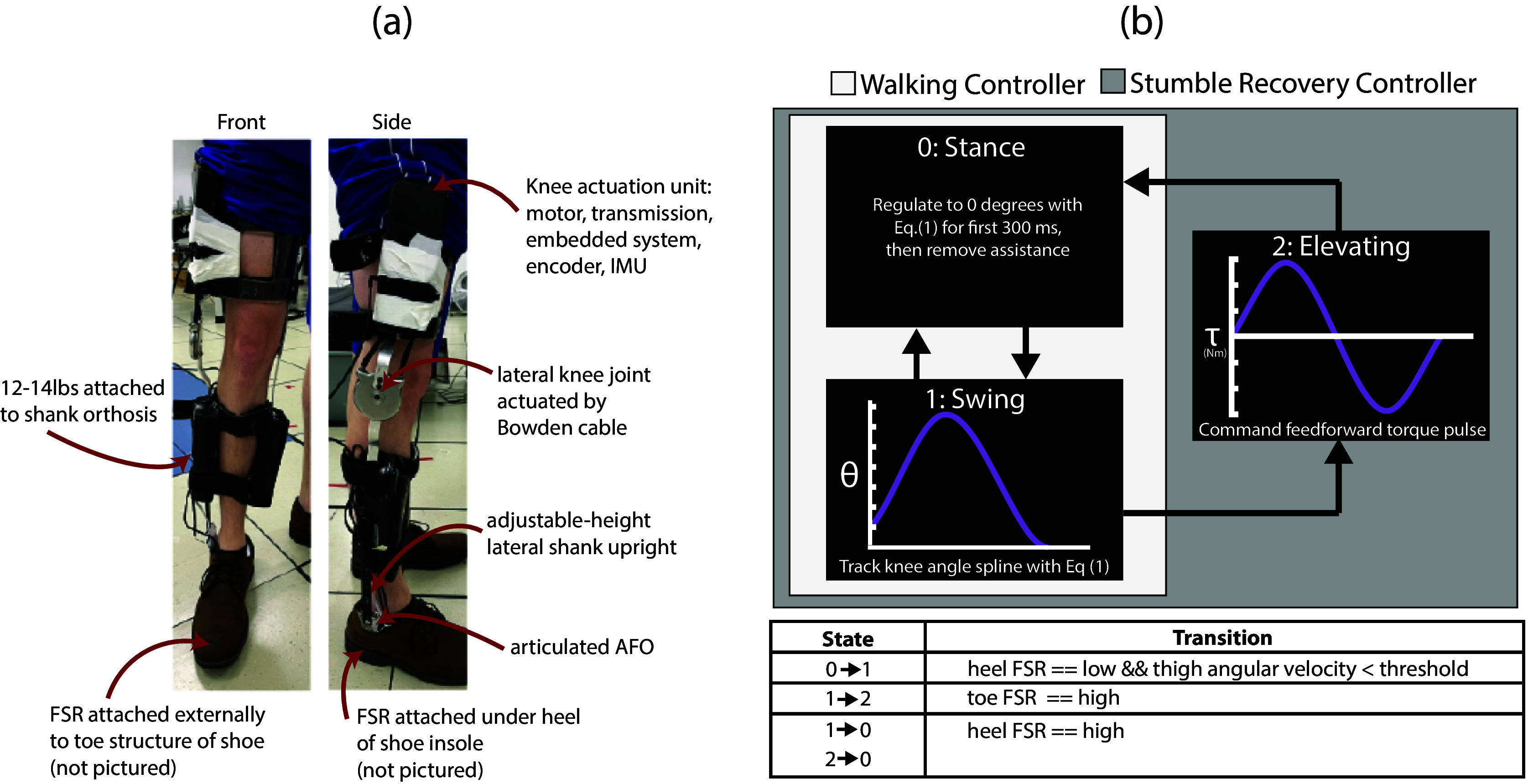
Interventional device and controllers. (a) A modified unilateral knee exoskeleton is used as the impairment method (leg weights attached to shank segment) and improvement method (powered knee module for flexion/extension assistance). (b) A walking controller and stumble recovery controller were designed to assist the knee joint during level ground walking and assist the knee in the execution of the elevating strategy, respectively.

A modified unilateral powered knee exoskeleton (Figure 1(a)) was used as both the impairment technique and the improvement technique in this study, discussed subsequently. The exoskeleton is a standard knee-ankle-foot orthosis (KAFO) modified with a non-standard powered knee module, which is actuated by a pair of Bowden cables driven by a brushless motor and a two-stage chain-drive transmission with a transmission ratio of 11:1. The transmission, embedded system, and sensors (nine-axis inertial measurement unit [IMU] to measure thigh motion and encoder to measure knee motion) are housed in a cassette attached to the thigh segment. Two force-sensing resistors (FSRs) are attached to the foot: one under the heel of the shoe insole (for swing/stance determination), and one externally on the toe structure of the shoe (for stumble detection). For purposes of the experiments conducted here, the device was powered with an off-board linear power supply (Kepco BOP36-12 M) that supplied up to 10A of current, which corresponded to 14.5 Nm of joint torque. An embedded system runs all low-level control, including brushless motor current control, and also includes a CAN interface that enables high-level control prototyping and data collection from a laptop computer via the real-time interface provided by MATLAB/Simulink at a sampling rate of 1 kHz. Thigh angular velocity is measured from the IMU with a first-order low-pass filter with a cutoff frequency of 10 Hz. Thigh angle is calculated via a standard complementary filter sensor fusion that combines measurements from the accelerometer and gyroscope, with a crossover frequency between the two measurements of 0.5 Hz.

#### 2.1.1. Impairment method

To impair walking gait as well as the elevating limb recovery response, 12–14 lb (5.4–6.4 kg) leg weights were attached to the shank segment of the knee exoskeleton, as pictured in Figure 1(a). Methods for evaluating the efficacy of these weights in impairing walking gait and stumble recovery are discussed in the Data Analysis subsection.

#### 2.1.2. Improvement method

A closed-loop walking controller was designed to assist the knee joint during level ground walking (Figure 1(b)), with the intent of correcting or reducing the simulated impairment introduced by the leg weights. The controller is comprised of a finite-state machine that transitions between two states: stance and swing. To prevent knee buckling during the initial stance, knee angle is regulated to zero degrees (set as the angle when the participant was standing upright with legs straightened, which was reset before each trial) using the following PD controller:
(1)

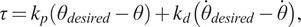

where 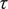 is the commanded torque, 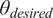 is 0 deg, 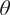 is the current knee angle, 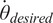 is 0 deg/s, 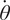 is current knee angular velocity, and 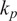 and 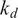 are experimenter-defined proportional and derivative gains, respectively. This assistance is removed 300 ms into stance to facilitate the initiation of swing phase by allowing knee flexion. To assist swing-phase motion, a position controller tracks a predefined knee angle trajectory (shown in Figure 1(b)) using Eq. 1, where 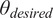 and 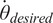 are the desired knee angle and knee angular velocity from the spline and its derivative at the current time in swing phase. The elapsed time of swing state is determined by a swing-stance ratio and the stance time of the current stride. State transitions are determined by heel FSR loading and thigh angular velocity thresholds (Figure 1(b)). The following variables were tunable for each user: 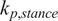 , 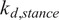 , 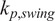 , 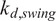 , peak swing knee angle, swing-stance ratio, and thigh angular velocity threshold.

A stumble recovery controller was designed to assist the knee in the execution of the elevating strategy. Upon detection of a stumble indicated by contact with the obstacle recorded from the toe FSR, a feedforward torque pulse intended to assist the flexion and subsequent extension of the elevating response is implemented. Specifically, the pulse is a 450-ms (approximate length of elevating strategies from a previous stumble study conducted at the same walking speed (King et al. (2019))) spline with approximately 14.5 Nm peak torque in flexion and extension. This spline is shown in Figure 1(b), in which the flexion torque and extension torque each span the same duration (225 ms). Note that if the participant took longer than 450 ms after perturbation to foot-strike, the controller commanded zero torque until foot-strike; if the participant’s foot-strike occurred before 450 ms, then the controller entered Stance Mode, as shown in the finite-state machine in Figure 1(b).

### 2.2. Stumble recovery experiment

#### 2.2.1. Experimental protocol

Three young, healthy adult participants (one female, two males; mean height; 1.77 m, mean mass: 77.9 kg; mean age: 26 years) were recruited to participate in the exoskeleton stumble recovery experiment. Participants were screened with the following inclusion criteria: Does weight added to the shank impair gait in a manner reflective of a target population, and does a knee exoskeleton walking controller improve the impaired gait? The first inclusion criterion was used to identify candidates who might simulate an impaired population. The second criterion was used to identify candidates whose simulated impairment could be corrected with the torque limits of the device. Therefore, the intent of this screening was to ensure that the participants started with a (steady-state) walking baseline that was representative of the set of individuals who would use a lower-limb exoskeleton for walking; namely, they have (1) an impaired gait without an exoskeleton and (2) a less impaired gait when using an exoskeleton. Quantitative measures to define this inclusion criterion are given in the Data Analysis subsection.

The protocol involved sessions on two separate days, an acclimation day and a testing day. All experimental protocols were approved by the Vanderbilt Institutional Review Board, and all participants gave their written informed consent.

During the acclimation day, the knee exoskeleton was fit to the participant’s right leg. Specifically, a thigh segment, shank segment, AFO, and shoe were selected that best fit the participant, and the height of the lateral shank upright was adjusted such that the center of the exoskeleton knee joint best approximated the axis of rotation of their biological knee. Then the participant walked on the treadmill at 1.1 m/s while the experimenter tuned the walking controller. Finally, the participant was introduced to a few stumbles (with the same setup and protocol discussed subsequently, including sensory occlusion techniques) in order to be familiarized with the experimental protocol prior to testing day.

**Figure 2.**
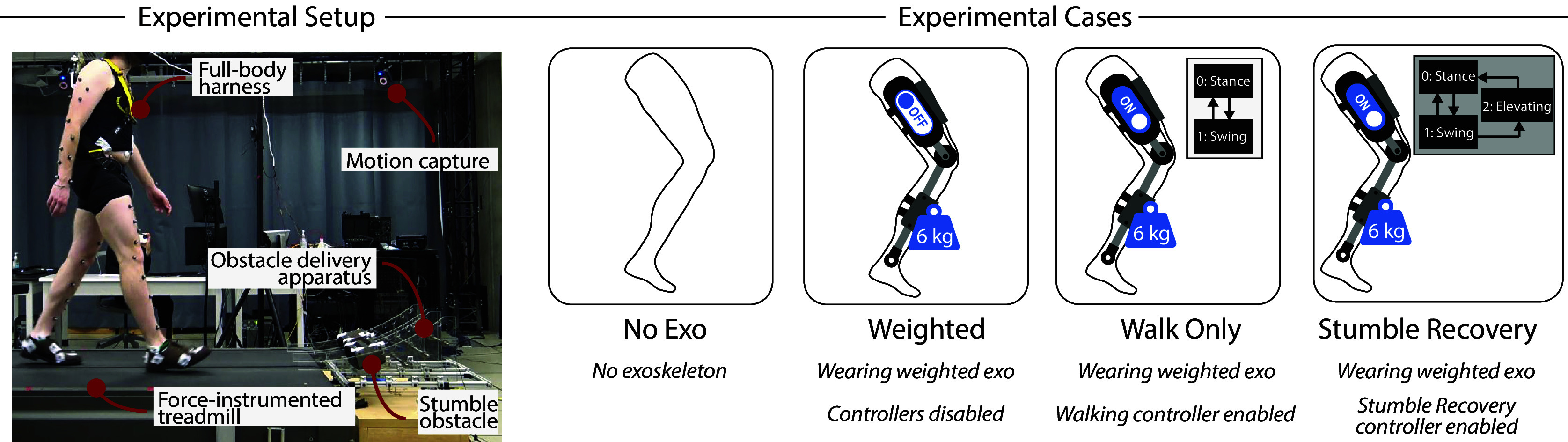
Stumble experiment setup and protocol. The obstacle stumble perturbation system described by King et al. (2019) is employed here (left). Stumbles trials were conducted for four experimental cases (right).

On testing day, the participant underwent a series of stumble trials using a custom obstacle perturbation system (King et al. (2019)). The experimental setup and protocol are pictured in Figure 2. For each trial, participants walked on a force-instrumented treadmill at 1.1 m/s. After a random number of strides (between 25 and 45), a 35 lb (16 kg) steel block was released from the obstacle delivery apparatus onto the treadmill belt such that it contacted the participant’s foot at an experimenter-defined percentage of swing phase. Participants were instructed to try to recover when the perturbation occurred and return to steady-state walking, after which the treadmill would be decelerated to end the trial. Therefore, each trial took between 30 s and 2 min, depending on the random number of strides before the stumble. Note that various sensory occlusion techniques (e.g., noise-canceling headphones and inferior vision-blocking goggles) and a cognitive distraction task (Serial Sevens) were employed to limit the expectation of the stumble. Additionally, the obstacle delivery apparatus was shown to deliver the obstacle without the perception of the participants in a previous validation study. See King et al. (2019) for full protocol and system details. For this experiment, perturbations were targeted to occur in the early swing phase (20–40% swing), such that elevating strategies would be employed to recover. Handrails were removed from the treadmill so they could not be used to recover, but participants wore a full-body harness to prevent contacting the treadmill in the event of a fall.

The stumble perturbations were introduced for four experimental cases, shown in Figure 2: (1) without wearing the exoskeleton (No Exo); (2) wearing the weighted-shank exoskeleton with the controllers disabled (Weighted); (3) wearing the weighted-shank exoskeleton with the walking controller enabled (Walk Only); and (4) wearing the weighted-shank exoskeleton with the stumble recovery controller enabled (Stumble Recovery). The No Exo case was intended to capture how an unimpaired, young, healthy adult responds to early swing perturbations, which served as the healthy control response. The Weighted case was intended to capture the response of the participant impaired by the weighted-shank exoskeleton without device assistance, which served as the baseline response of the impaired individual. The Walk Only case was intended to capture how an impaired individual responded if they were wearing an exoskeleton that assisted walking but did not have a stumble recovery feature. Finally, the Stumble Recovery case was intended to capture how an impaired individual responded if they were wearing an exoskeleton that assisted walking and also provided stumble recovery assistance when detected. Note that for both the Stumble Recovery and the Walk Only cases, weights were still attached to the shank.

The No Exo trials were conducted first. The order of exoskeleton trials (Weighted, Walk Only, Stumble Recovery) was randomized. Additionally, trials in which the left limb (i.e., the limb not wearing the exoskeleton) was stumbled were interspersed randomly; these trials were included so that not all trips would occur on the right side, in order to limit expectation of the stumble event and any anticipatory behaviors associated with knowing only the right side could be tripped. These trials were not included in the analysis, as the exoskeleton controller tested in this work was designed for the elevating limb. The participant was not informed of the case of each trial. Each participant was introduced to 3–6 trials for each case. Additionally, before and after the stumble perturbation trial sets, 60-s walking trials (without perturbation) were conducted for the No Exo, Weighted, and Walk Only cases.

#### 2.2.2. Data collection & processing

For each trial, ground reaction forces (GRFs) were collected at 1,000 Hz via a lateral, split-belt, force-instrumented treadmill (Bertec, Columbus, USA). Full-body kinematic data were collected at 200 Hz via infrared motion capture (Vicon, Oxford, GBR), which included feet, shank, thigh, pelvis, trunk, forearm, and upper arm segments. GRF and motion capture data were filtered with a zero-phase, third-order, low-pass Butterworth filter with cutoff frequencies of 15 and 6 Hz, respectively. Inverse kinematics were computed using Visual3D (C-Motion, Germantown, USA) to estimate joint-level kinematics for each trial.

#### 2.2.3. Data analysis

Trials were excluded from the analysis based on a few criteria. First, if the participant stepped on the obstacle or over the obstacle (i.e., missed targeting), the trial was not deemed a swing-phase perturbation and was excluded. Second, if the participants’ foot hit the obstacle substantially medially, this caused the block to rotate which is an outlier relative to remaining stumbles (i.e., perturbation impulse is altered, and in some cases block rotated enough such that it did not need to be cleared); thus, if the block rotated more than 45 degrees about the vertical axis after contacting the participants’ foot, the trial was excluded from the analysis.

For each trial, the perturbation event was identified as the instant at which the foot contacted the obstacle, which was determined via a transient peak in the anterior–posterior GRF measured by the treadmill. The swing percentage of each perturbation was estimated as the time from the toe-off immediately preceding the perturbation to the instant of perturbation, relative to the average of 20 swing times prior to the perturbation.

To determine participant inclusion in the experiment, (i.e., participants for whom adding unilateral leg weights would approximate the gait of an impaired population, and for whom the exoskeleton could correct this impaired gait), overall temporal symmetry was computed using gait information from the average of 20 strides during the walking trials for each case. Overall temporal symmetry is defined here as the ratio of the right limb’s temporal swing-stance symmetry to the left limb’s temporal swing-stance symmetry (Patterson et al. (2008)). Temporal swing-stance symmetry is defined as the ratio of swing time to stance time for a given limb, where swing time is the time from toe-off to foot-strike, and stance time is the time from foot-strike to toe-off. In a study examining the gait asymmetry of community-ambulating stroke survivors, the normative range for overall temporal symmetry was defined as 0.9 to 1.1, mild asymmetry was defined to be 1.1 to 1.5, and severe asymmetry defined as greater than 1.5 (with a value over 1.0 indicating a preference to rely on the non-paretic limb) (Patterson et al. (2008)). Note that gait asymmetry has been identified as a factor for predicting falls for stroke patients (Wei et al. (2017)). These ranges were used to determine if the participants were adequate candidates for the impairment and improvement techniques chosen for this study to represent an impaired population who might use a lower-limb exoskeleton. Specifically, if the Weighted case induced an asymmetry in the mild range, and the asymmetry was restored to the normative range with the Walk Only case, then the participant’s gait (1) was considered to be substantially impaired with the weighted-shank exoskeleton, and (2) could be corrected with the torque assistance available from the current device. Note that half of the individuals screened for this inclusion criterion passed. Two participants did not pass the first criterion when screened, as they forcibly overcame the inertial asymmetry imposed by the leg weights following acclimation. One participant just passed the first criterion, but did not pass the second, likely due to exoskeleton torque limitations.

**Figure 3.**
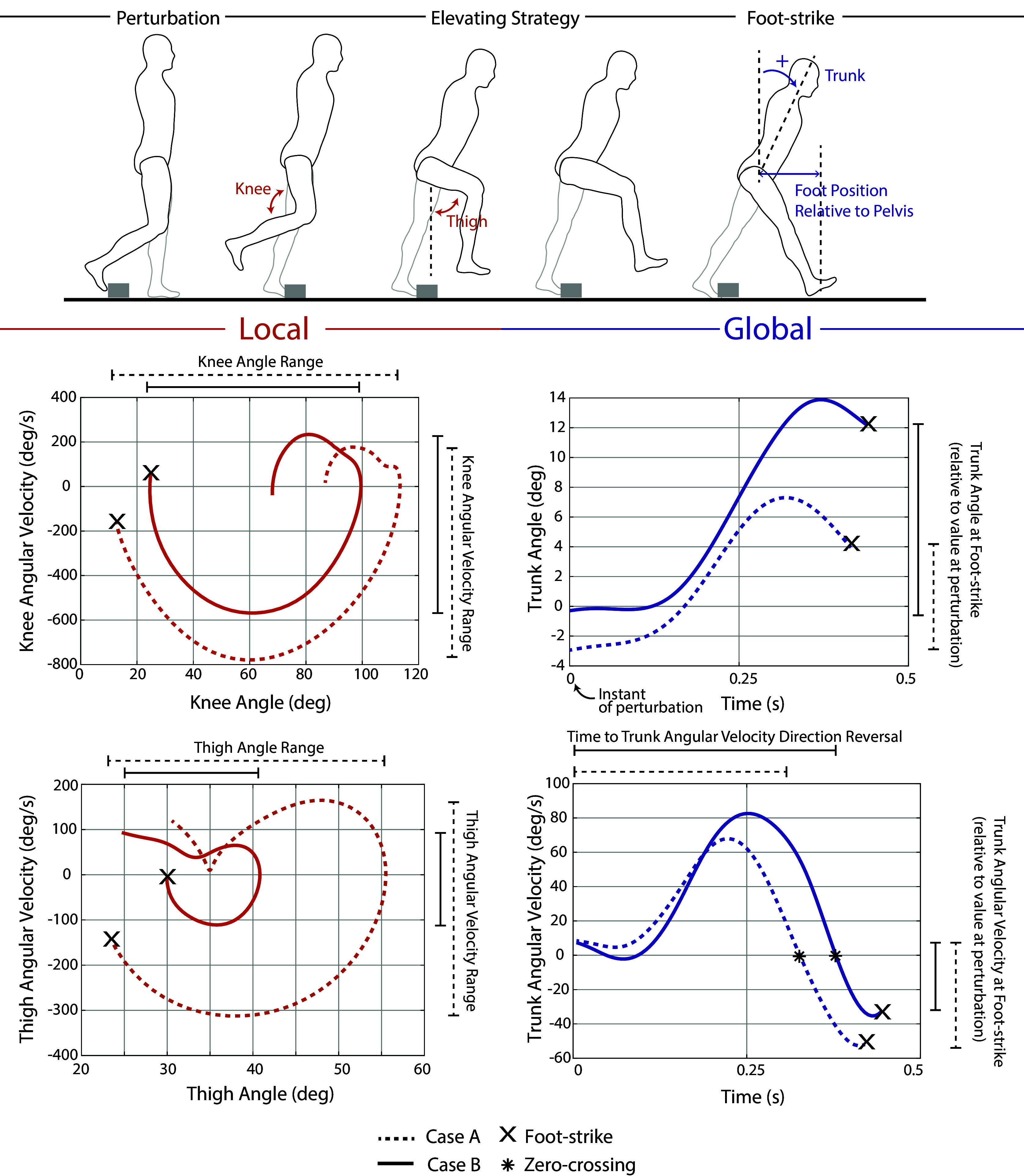
Outcome metrics to assess local and global impairment/improvement. Data from a representative comparison pair (perturbations at similar swing percentages from Case A and Case B) are plotted. The difference in each metric (Case A relative to Case B) was computed for each comparison pair for analysis in Figures 5, 6, and 7. Specifically, for Figure 5, Case A is Weighted and Case B is No Exo; for Figure 6, Case A is Stumble Recovery and Case B is Weighted; for Figure 7, Case A is Stumble Recovery and Case B is Walk Only.

Various outcome metrics were computed to assess the extent to which the weighted-shank exoskeleton and stumble recovery controller respectively impaired and improved the participants’ stumble recovery response. Both local (elevating limb kinematics) and global (overall fall risk) metrics were considered. These outcome metrics are diagrammed in Figure 3 along with representative trajectories. To evaluate local impairment/improvement, the range in knee angle, knee angular velocity, thigh angle, and thigh angular velocity from the instant of perturbation to the first foot-strike of the recovery limb were computed for each trial. To evaluate global impairment/improvement, trunk angle and trunk angular velocity at recovery foot-strike were computed (calculated relative to the value at perturbation). Additionally, the elapsed time from perturbation to the instant at which trunk angular velocity reversed direction from forward rotation to backward rotation (i.e., how soon induced forward angular momentum was arrested) was computed. These trunk metrics reflect the participants’ ability to restore trunk motion during the recovery. Finally, the recovery foot’s anterior–posterior center-of-mass (COM) position relative to the pelvis’ anterior–posterior COM position at recovery foot-strike (calculated relative to the value at perturbation) was computed for each trial. Trunk angle, trunk angular velocity, and foot positioning at recovery foot-strike have previously been reported to discriminate falls from recoveries (Pavol et al. (2001); Owings et al. (2001); Grabiner et al. (2012); Pater et al. (2016)) and many works cite trunk control and foot positioning as keys to successful recovery (Grabiner et al. (1993); Eng et al., 1994; Schillings et al. (2000); Forner Cordero et al. (2003); Pijnappels et al. (2005a)); thus changes in these metrics can be used as indicators of fall risk or improved recovery.

To examine the extent of stumble recovery impairment, the local and global outcome metrics from Weighted trials were compared to No Exo trials. Since stumble recovery responses vary depending on when in swing phase the perturbation occurs (Eng et al. (1994); Schillings et al. (2000); Shirota et al. (2014b); Eveld et al. (2021b)), it was important to compare outcome metrics from like perturbations (i.e., occurring at the same swing percentage, or as close as possible) in order to interpret the effect of the intervention with other perturbation factors controlled. Thus, trials that were closest in swing percentage were paired using the following procedure: each successful Weighted trial was paired with a No Exo trial that was closest in swing percentage. If there were no No Exo trials within 3% swing of said Weighted trial, then that Weighted trial was not used in the analysis. Conversely, if there were more than one No Exo trial within 1% swing of said Weighted trial, then both (or more) No Exo trials were paired with the Weighted trial to comprise two (or more) comparison pairs. For each comparison pair, the difference in each outcome metric (Weighted relative to No Exo) was computed.

To examine the extent of stumble recovery improvement, the identical comparison pair approach was used for (i) Stumble Recovery relative to Weighted and (ii) Stumble Recovery relative to Walk Only.

## 3. Results

**Figure 4.**
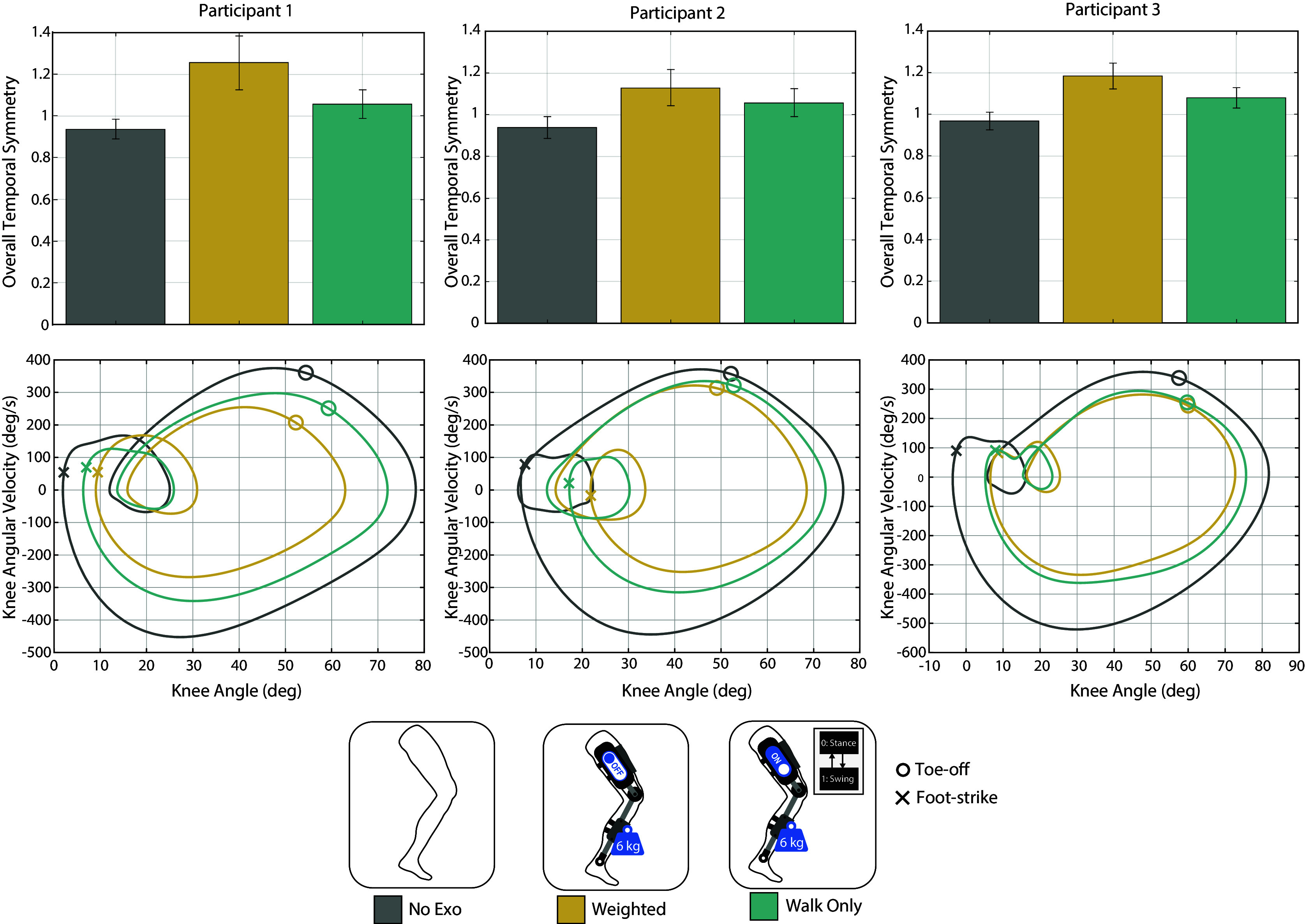
Overall temporal symmetry and knee motion for walking trials for each participant.

The weighted-shank exoskeleton substantially impaired walking gait, and this impairment was restored or considerably improved with the walking controller for the three participants. The overall temporal symmetry, as well as the phase plane of knee angle versus knee angular velocity for each participant for each case (No Exo, Weighted, and Walk Only), is shown in Figure 4. The overall temporal symmetry for the three participants who did not pass the inclusion criterion is given in Supplementary Material Figure S1.

**Figure 5.**
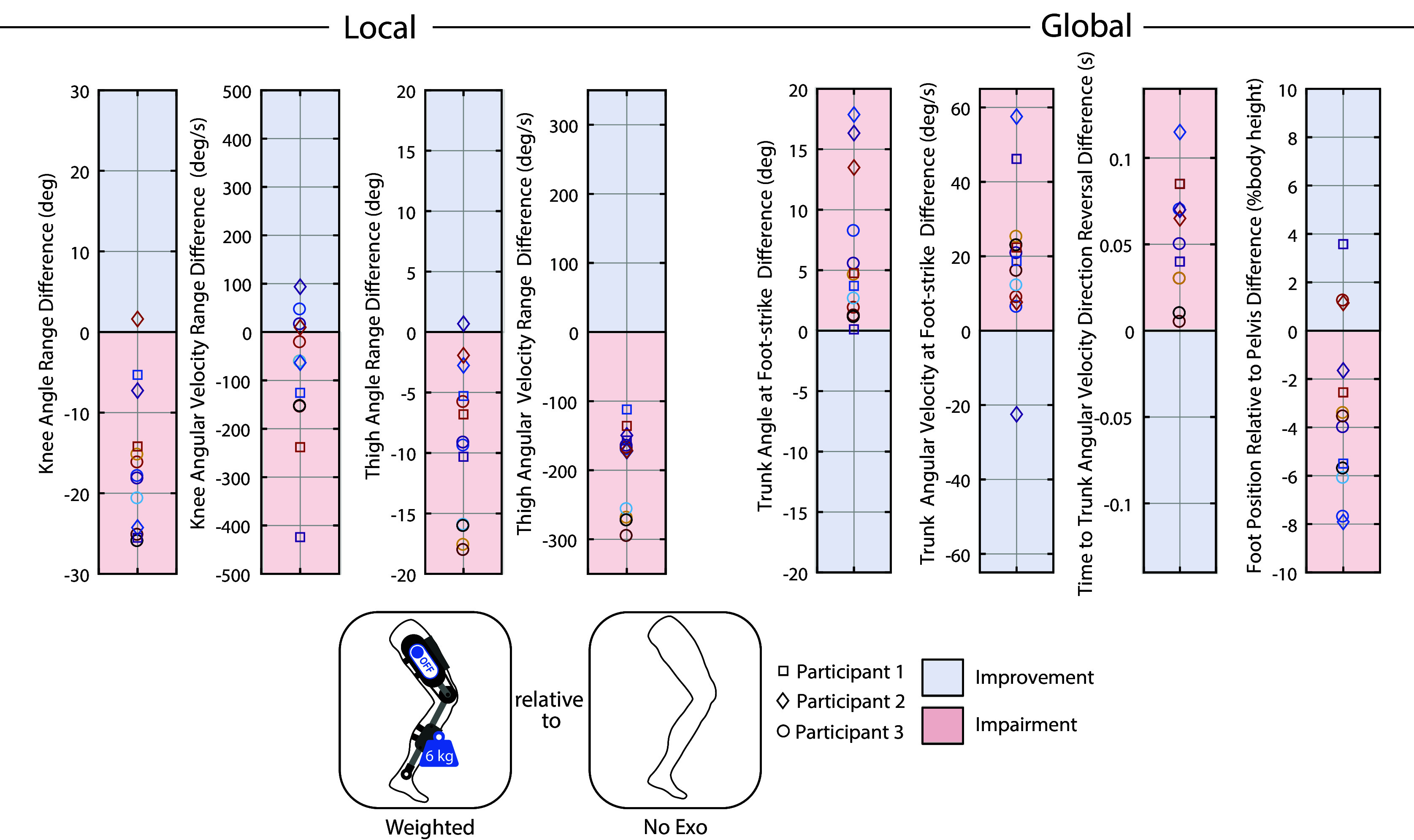
**Weighted relative to No Exo** Each marker represents a comparison pair (i.e., a Weighted and No Exo stumble that occurred at the same swing percentage) and plots the difference in the indicated metric between the two responses. See Figure 3 for how each metric was calculated. Differences that are considered improvements are shaded in blue, while impairments are shaded in red. For example, the bottom-most circle marker in the Knee Angle Range Difference plot indicates that Participant 3’s knee angle range during a Weighted elevating response was 28 degrees less than their knee angle range during a No Exo elevating response from a stumble at the same swing percentage, which is considered a local impairment. Likewise, Participant 3 landed their elevating recovery step with 22 deg/s more trunk angular velocity (black circle in Trunk Angular Velocity plot), which is considered a global impairment. Overall, leg weights attached to the shank impaired the elevating limb response (local) and increased fall risk (global) relative to responses when not wearing the leg weights.

**Figure 6.**
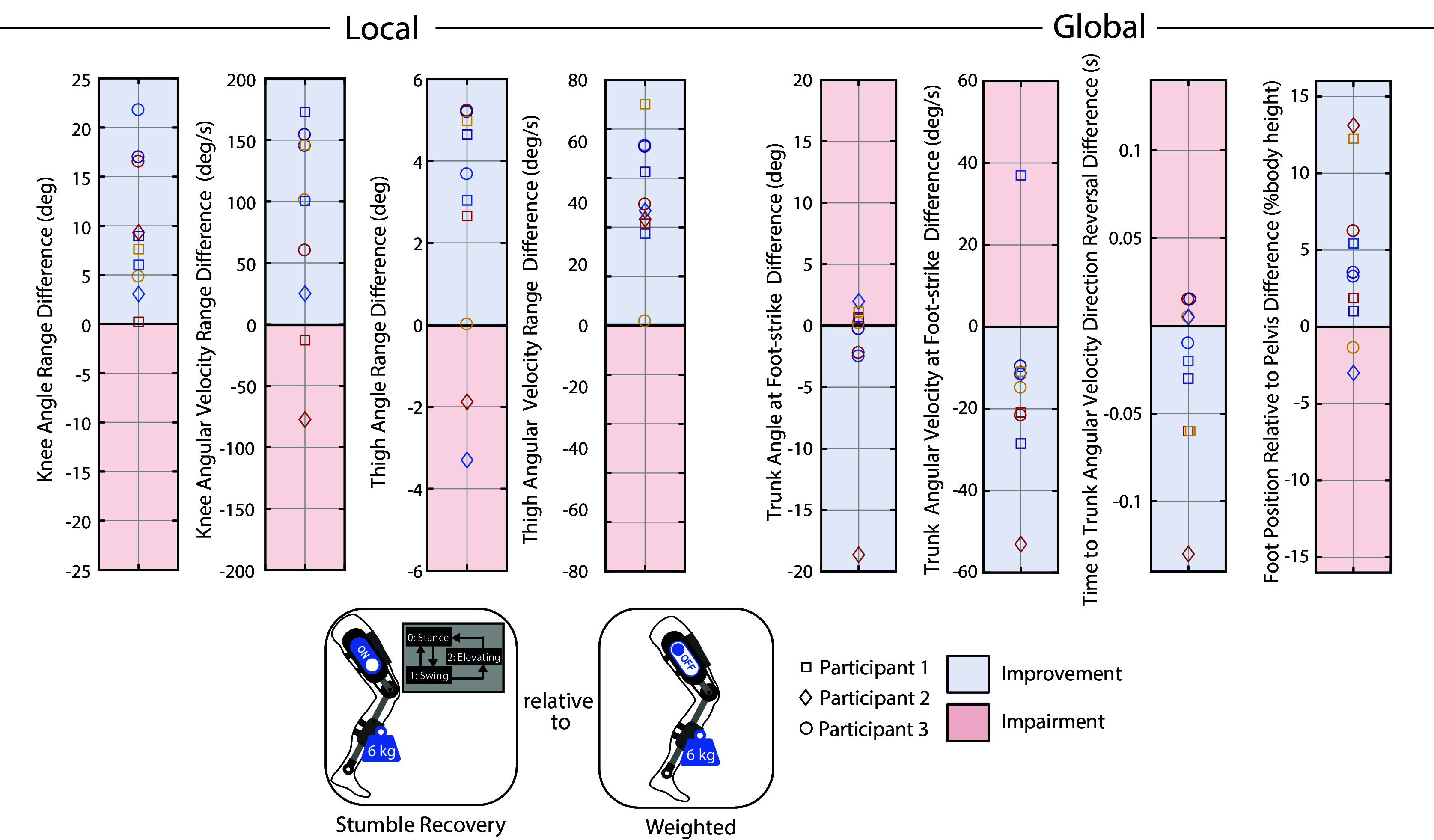
**Stumble Recovery relative to Weighted** Each marker represents a comparison pair (i.e., a Stumble Recovery and Weighted stumble that occurred at the same swing percentage) and plots the difference in the indicated metric between the two responses. See Figure 3 for how each metric was calculated. Differences that are considered improvements are shaded in blue, while impairments are shaded in red. For example, the top-most circle marker in the Knee Angle Range Difference plot indicates that Participant 3’s knee angle range during a Stumble Recovery elevating response was 23 degrees more than their knee angle range during a Weighted elevating response from a stumble at the same swing percentage, which is considered a local improvement. Likewise, Participant 3 landed their elevating recovery step with 10 deg/s less forward trunk angular velocity (blue circle in Trunk Angular Velocity plot), which is considered a global improvement. Overall, the stumble recovery assistance improved the elevating limb response (local) and reduced fall risk (global) relative to responses when impaired with leg weights without assistance.

**Figure 7.**
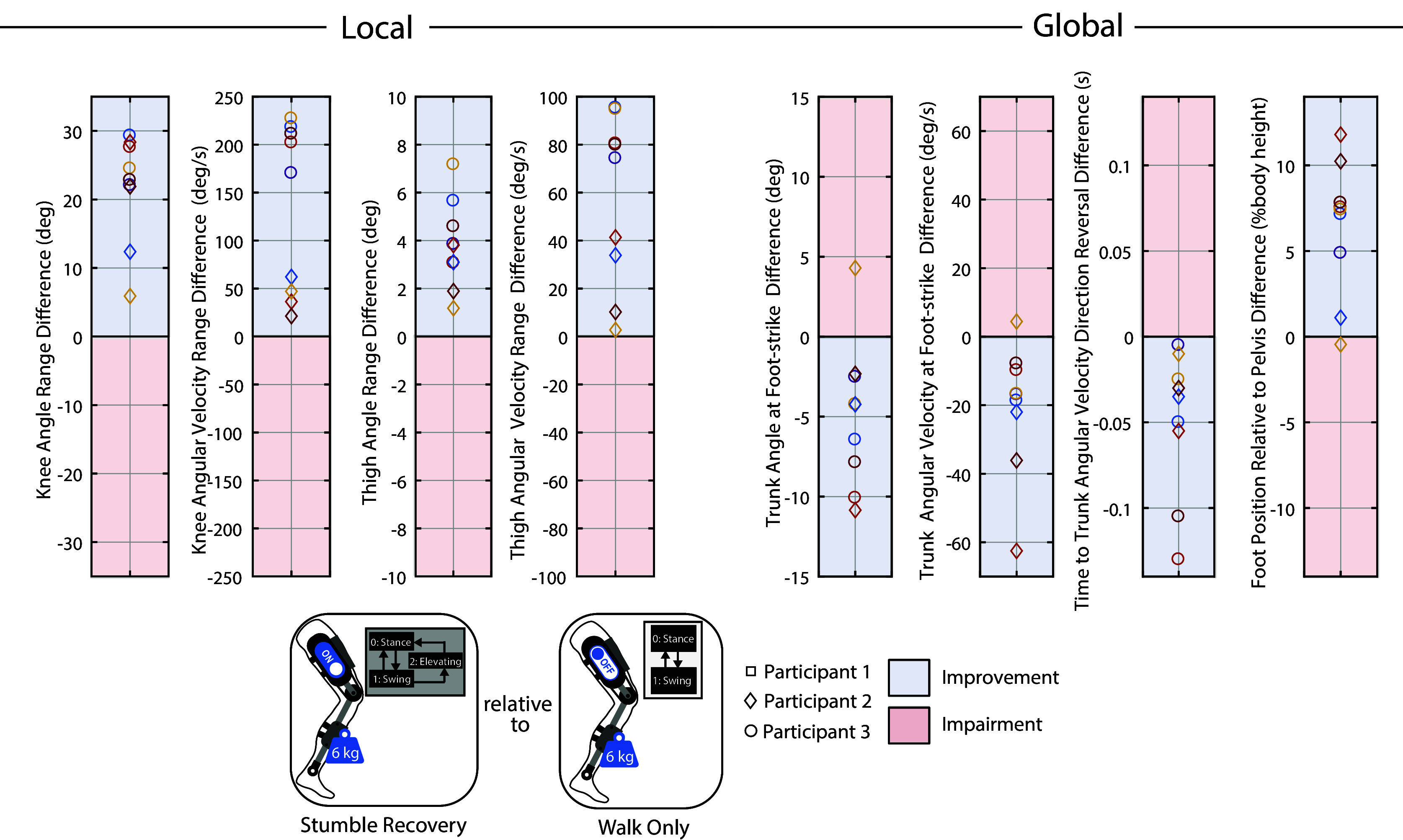
**Stumble Recovery relative to Walk Only** Each marker represents a comparison pair (i.e., a Stumble Recovery and Walk Only stumble that occurred at the same swing percentage) and plots the difference in the indicated metric between the two responses. See Figure 3 for how each metric was calculated. Differences that are considered improvements are shaded in blue, while impairments are shaded in red. For example, the top-most circle marker in the Knee Angle Range Diference plot indicates that Participant 3’s knee angle range during a Stumble Recovery elevating response was 30 degrees more than their knee angle range during a Walk Only elevating response from a stumble at the same swing percentage, which is considered a local improvement. Likewise, Participant 3 landed their elevating recovery step with 19 deg/s less forward trunk flexion velocity (blue circle in Trunk Angular Velocity plot), which is considered a global improvement. Overall, the stumble recovery assistance improved the elevating limb response (local) and reduced fall risk (global) relative to responses when impaired with leg weights with only walking assistance.

After removing trials due to (1) the participant stepping on the obstacle, (2) the obstacle rotating greater than 45 degrees about the vertical axis, or (3) the trial timing being deemed unsuitable for comparison to other trials (i.e., no other trials within 3% swing), 37 trials were available for analysis. Supplementary Material Figure S2 maps comparison pairs for each participant, according to the procedure outlined in Methods. Figures 5, 6, and 7 plot the difference in local and global outcome metrics for Weighted relative to No Exo comparison pairs (i.e., assessing impairment), Stumble Recovery relative to Weighted comparison pairs (i.e., assessing improvement), and Stumble Recovery relative to Walk Only comparison pairs (i.e., assessing improvement). Impairments were determined by less knee angle/angular velocity range, less thigh angle/angular velocity range, less anterior foot position relative to pelvis (i.e., negative difference) and more trunk angle, more trunk angular velocity, and longer time to trunk angular velocity reversal (i.e., positive difference), which are indicated by red shading in Figures 5, 6, and 7. Blue shading indicates improvement (positive difference for knee, thigh, and foot position metrics, and negative difference for trunk metrics).

## 4. Discussion

### 4.1. Assessment of gait impairment and improvement: Weights attached to shank impair gait, exoskeleton walking controller improves it

Weights attached to the shank segment of the exoskeleton substantially impaired the three participants’ walking gait in terms of temporal asymmetry and knee motion. The weights increased participants’ right-side swing time and thus increased their right-side swing-stance ratio, as participants took longer to move their right limb due to its added mass. As shown in Figure 4 (top), this resulted in increased overall asymmetry values for the Weighted case (all in the mild asymmetry range) compared to their No Exo trials (all in the normative asymmetry range). Furthermore, these Weighted overall temporal asymmetry values reached levels akin to the mild asymmetry experienced by stroke survivors, and thus may approximate gait abnormalities exhibited by those at higher fall risk (Patterson et al., 2008; Wei et al., 2017). Additionally, the added weight to the participants’ shank segment hindered their ability to flex and extend their knee joint, as evidenced by a decrease in knee motion (angle and angular velocity) for the Weighted case relative to No Exo (Figure 4, bottom).

The exoskeleton walking controller improved the walking gait (symmetry and knee motion) for the three participants. The trajectory-tracking controller provided the assistance to complete a faster (more symmetrical) swing time. Specifically, flexion assistance at toe-off helped initiate swing phase and reach more normative knee flexion during swing, while the extension assistance helped to complete swing phase in a timely manner. While overall temporal asymmetry was not fully restored to No Exo values, it was reduced for each participant below the mild asymmetry threshold of 1.1 to the normative range (Figure 4, top). Similarly, knee range metrics for the Walk Only case increased relative to Weighted, though not to No Exo values (Figure 4, bottom). A knee exoskeleton with greater control authority may have served to fully restore symmetry and knee motion (i.e., the device used here was torque-limited, particularly relative to the increased leg inertia associated with the added leg weights).

Note that in the screening process for participant inclusion, two participants did not pass the first criterion (i.e., did not show gait impairment). This may be due to differences in muscle strength and/or level of comfort/relaxation during the experiment. One participant did not pass the second criterion (i.e., did not show gait improvement). This may be due to level of comfort/relaxation during the experiment and/or the torque limitations of the exoskeleton device.

These outcomes were important validations that weights attached to the shank segment of an exoskeleton could reasonably approximate impaired gait, and that an exoskeleton knee controller could improve it, which were important preliminary validations for the stumble assessment. However, these walking outcomes do not assess the extent to which weights might impair a stumble response, or the extent to which an exoskeleton might improve it.

### 4.2. Assessment of stumble recovery impairment: Weights attached to shank impair elevating limb response and increase fall risk

Overall the recovery limb was substantially impaired when elevating over the obstacle due to the added weight at the shank. This was evidenced by comparing the elevating kinematics of the participants’ right limbs when wearing the weighted exoskeleton (Weighted) to their kinematics when not wearing an exoskeleton (No Exo). 12 of 13 comparison pairs (see Methods) exhibited a decrease in knee and thigh range of motion during the elevating response for the Weighted case relative to No Exo. Nine pairs exhibited a decrease in knee velocity range, and all pairs showed a decrease in thigh velocity range (Figure 5).

However, an intervention that impairs locally (where the intervention was applied) does not necessarily imply that the individuals’ overall response was impaired. Thus, it was important to also consider global response outcome metrics. As shown in Figure 5, the participants landed their elevating step with more trunk flexion (all pairs) and more trunk flexion velocity (12/13 pairs) in the Weighted case relative to their No Exo trials. In previous laboratory-induced tripping studies, increases in these two metrics have repeatedly been reported as discriminators between those who fall and those who recover (Pavol et al., 2001; Grabiner et al., 2012; Pater et al., 2016). Furthermore, for all pairs, the participants reversed their trunk velocity direction (from forward rotation to backward rotation) later (i.e., longer elapsed time after perturbation) for the Weighted case relative to the No Exo case. This indicates a delayed ability to control trunk motion and arrest forward momentum, also suggesting a higher risk of falling. Finally, for most trials (10/13) the participants landed with their foot less anterior to their pelvis compared to the No Exo case. Previous studies report foot positioning as a key factor in a successful recovery (Grabiner et al., 2012; Pater et al., 2016; Owings et al., 2001; Crenshaw et al., 2012; Honeycutt et al., 2016) (i.e., more anterior foot position is better).

Therefore, the change in local outcome metrics indicates a deficient elevating limb response, and the change in global outcome metrics indicates that this local impairment extended to an overall increase in fall risk. These results were important for ultimately addressing the main objective – Can knee exoskeleton stumble recovery assistance improve an impaired stumble recovery response? Additionally, these results further bolster the hypotheses from prior studies that the recovery limb plays a crucial role in recovery (Pijnappels et al., 2005a; Grabiner et al., 1993; Forner-Cordero et al., 2014) by showing that its impairment increases fall risk.

### 4.3. Assessment of stumble recovery improvement: Exoskeleton stumble recovery controller improves elevating limb response and reduces fall risk

Ultimately, the objective of this work was to determine the extent to which a powered knee exoskeleton stumble recovery controller could improve an impaired stumble recovery response. This question was assessed by comparing the participants’ responses to perturbations with the stumble recovery controller enabled (Stumble Recovery) to their Weighted (i.e., impaired) response. For all 10 comparison pairs, the participants’ knee range of motion during the elevating response increased when the stumble recovery controller was enabled, and knee velocity range increased for eight of those pairs (Figure 6). Thus the knee flexion/extension torque served to successfully assist the knee, providing a response more akin to their No Exo trials. Furthermore, thigh range of motion and thigh velocity range also increased for 8/10 and 10/10 pairs, respectively. Thus this dedicated stumble recovery assistance at the knee not only improved knee motion but also facilitated a better thigh response, both of which are key components of the elevating step (Grabiner et al., 1993; Eveld et al., 2022).

While previous works have suggested improving the elevating step as a promising target for intervention, it was important to confirm that improvement at the local level (at the site of intervention) also extended to global improvement (i.e., decreased fall risk). As shown in Figure 6, for most comparison pairs the participants landed the elevating step with decreased trunk velocity (9/10 pairs) and a more anterior foot position relative to their pelvis (8/10 pairs), both of which indicate a decrease in fall risk (as discussed previously). Additionally, participants reversed their trunk velocity direction sooner relative to the Weighted case for 6/10 pairs, which indicates that they were able to arrest the perturbation-induced forward angular momentum (i.e., control trunk motion) more effectively with the stumble recovery controller enabled. The majority of cases did not see an improvement (decrease) in trunk flexion (4/10); however, note that trunk flexion did not increase more than three degrees, so the stumble recovery controller did not substantially increase this fall risk metric.

The Stumble Recovery responses were also compared to the Walk Only cases. This shows the difference in responses while wearing a rehabilitation exoskeleton (i.e., one that provides walking assistance) with versus without a stumble recovery feature. As shown in Figure 7, for all nine comparison pairs the responses with the Stumble Recovery controller exhibited increased knee and thigh angle and velocity ranges relative to the Walk Only cases. Recall that for the walking controller (Walk Only), a knee angle spline was tracked with a closed-loop PD controller in swing phase; thus, when a perturbation happened in swing (and was not detected by the controller), the participant attempted a knee trajectory different from the planned trajectory. Specifically, when the participant attempted to flex additionally after contacting the obstacle, the knee controller was commanding extension in the knee to complete swing phase. This mismatch in user intent and controller planning restricted the participant’s elevating limb kinematics, which ultimately increased the users’ fall risk. For most comparison pairs, the stumble recovery controller outperformed the walking controller in terms of fall risk, exhibiting less trunk flexion (8/9 pairs) and trunk flexion velocity (8/9) at foot-strike, a quicker reversal of trunk velocity direction (9/9), and a more anterior foot placement relative to the pelvis (8/9).

Therefore, the proposed knee exoskeleton stumble recovery controller successfully improved participants’ lower-limb response kinematics and consequently improved fall risk metrics relative to their Weighted responses and Walk Only responses.

This is the first study to show that stumble recovery assistance in a knee exoskeleton could improve an impaired stumble recovery response. This validation with healthy individuals now invites the testing of this approach with fall-prone populations, such as the elderly, stroke survivors, post-polio patients, or multiple-sclerosis patients, who may benefit from exoskeleton knee assistance. Such populations exhibit some of the impairments provided by the weighted-shank technique used here (i.e., gait asymmetry, decreased knee/thigh range of motion, decreased muscle response or delayed response time), and thus they may be good candidates for the proposed exoskeleton knee assistance. The authors note, however, that the impairment tested in this work is a coarse approximation of a neuromuscular deficit and fails to capture many aspects of various gait impairments (e.g., neurological impairment); furthermore, the healthy participants tested in this work have access to various response mechanisms that may not be available to some fall-prone populations. Therefore, future work is needed to test the extent to which this stumble recovery improvement approach may extend to fall-prone populations.

The authors also note that lower-limb exoskeletons are emerging not only as rehabilitation devices for individuals with walking impairment but also as augmentation devices for healthy individuals (e.g., Zoss et al. (2006)). Although tripping over obstacles is certainly an eventuality, such devices do not currently account for stumble perturbations. Without an incorporated stumble response, existing mechanical or control features of an exoskeleton (e.g., added weight, joint impedance, or restrictive controller) might limit the ability of the exoskeleton user to react adequately to an unexpected stumble. Since this work has shown the efficacy of knee assistance for stumble recovery for healthy individuals, and the impairment technique used in this work could also approximate the effect of a heavier exoskeleton, implementing this or a similar intervention into existing exoskeletons may reduce an individual’s fall risk while wearing a lower-limb exoskeleton.

### 4.4. Limitations

There are several limitations to this work. First, in this study the improvement intervention was limited by the specifications of the knee exoskeleton device available. The maximum knee torque for given recovery speeds was applied to the participants; however, a device with higher torque and speed capacity would likely have improved responses.

Second, in this work only one type of stumble recovery assistance was explored (i.e., a feedforward torque flexion-extension pulse upon stumble detection). Results proved that this assistance at the knee helps; however, future works could explore different types and levels of assistance to optimize response outcomes, such as assistance magnitude, timing, and control strategy.

Third, this work compared the Stumble Recovery case to both the Weighted and Walk Only cases to show Stumble Recovery improvement; however, note that the Walk Only case results are specific to the type of walking controller used, and as such other walking controllers may have yielded different results. For example, Akiyama et al. (2020) suggested that their walking controller improved healthy users’ stepping responses after tripping compared to stopping assistance after the perturbation; their walking controller only implemented feedforward torques that assisted hip flexion and knee extension in swing phase, as opposed to the trajectory-tracking controller used here. Still, the authors contend that a trajectory-tracking controller is a common form of walking controller for lower-limb exoskeletons to date, so it was reasonable to use it for the Walk Only case. Importantly, this case emphasizes the following point: Exoskeletons that ignore potential stumbles could be detrimental to the user, and therefore it is crucial for exoskeleton designers to account for stumbles in their controllers. Similarly, there are other behaviors that could be implemented after perturbation that were not tested in this work, and warrant further investigation.

Fourth, only stumble recovery responses using the elevating strategy were tested in this work. Efficacy for assistance during the lowering strategy (the other primary stumble recovery strategy) warrants exploration; however, the lowering strategy also involves quick knee flexion and extension of the recovery limb, and so a similar approach could be feasible. Note that accounting for lowering strategies would additionally require the decision between elevating versus lowering responses in the controller, which is crucial to be robust for all stumbles; such a strategy selection algorithm was explored in Eveld et al. (2023).

Fifth, there are other metrics that could have been used to quantify and/or provide context to impairment and improvement in stumble recovery. For example, a decrease in muscle effort during the response could be considered an improvement. Likewise, an analysis of arm movement could give perspective on additional compensatory strategies used to recover. Future work could examine the effect of exoskeleton assistance on these metrics as well, which would complement these findings.

Sixth, the tuning process for the walking controller was based on qualitative measures via an unstandardized (but informed) sweep of parameters, adjusting for user comfort and visual symmetry; a more controlled tuning approach could have offered insights into resulting outcome metrics. Additionally, participants had varying degrees of exoskeleton experience before the experiment, which could have affected responses.

Finally, more trials from more participants would further support these results; however, considering the taxing nature of this protocol, and that the same trends were seen across all three participants, the authors contend that these results provide the initial validation needed to confirm the promise of this intervention method and motivate the development of this approach for the applications discussed previously.

## 5. Conclusion

A knee exoskeleton stumble recovery controller successfully improved participants’ elevating stumble recovery responses at both the local level (i.e., improved recovery limb kinematics) and global level (i.e., decreased fall risk) in an obstacle perturbation experiment in which healthy participants were impaired (locally and globally) with weights attached to the shank. Ultimately, this initial exploration indicates that providing similar assistance to individuals with walking impairment might improve recovery responses and reduce the likelihood of falling, although that assertion must of course be substantiated with future work. Nonetheless, this preliminary work provides a crucial first step in investigating the potential efficacy of wearable lower-limb devices in stumble recovery roles, and substantiates the potential promise of such stumble recovery assistance in eventually enhancing the safety and adoption of lower-limb exoskeletons and mitigating falls in fall-prone populations.

## Supporting information

Eveld et al. supplementary material Eveld et al. supplementary material

## Data Availability

Data can be made available to interested researchers upon request by email to the corresponding author.

## Supplementary material

The supplementary material for this article can be found at http://doi.org/10.1017/wtc.2023.17.
