# Supplementary material for: Efficacy of stumble recovery assistance in a knee exoskeleton for individuals with simulated mobility impairment: A pilot study
Source: Wearable Technol. 2023 Sep 6;4:e22. doi: 10.1017/wtc.2023.17 (PMC10952054; doi:10.1017/wtc.2023.17)
Supplement: Eveld et al. supplementary material [file S2631717623000178sup001.pdf]

Supplementary Material corresponding to:

Efficacy of stumble recovery assistance in a knee exoskeleton for individuals with simulated mobility impairment: A pilot study

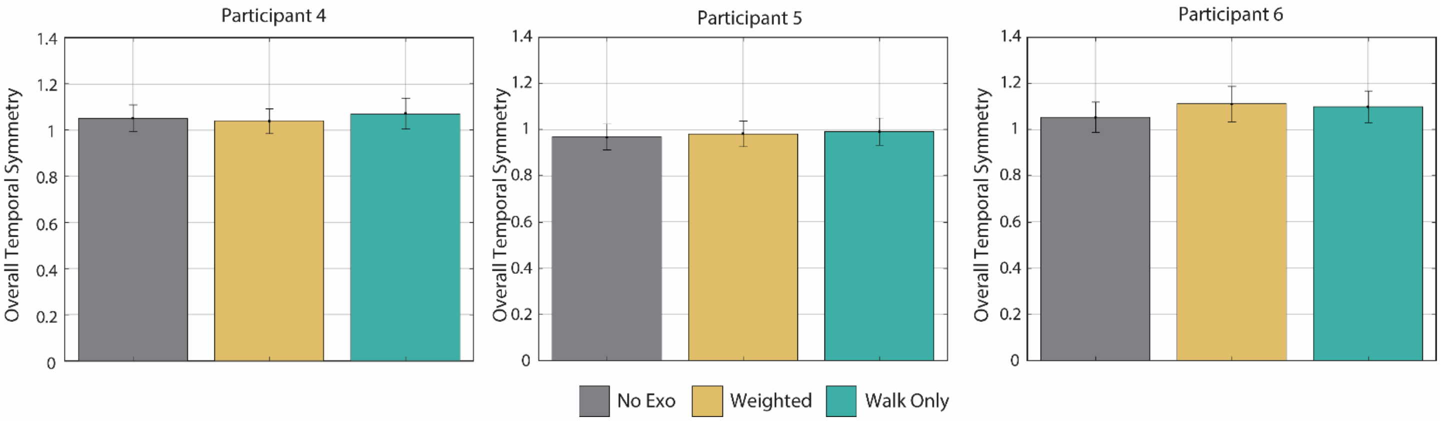

Figure S1. Overall Temporal Symmetry for participants who did not pass inclusion criterion. Participants 4 and 5 did not pass the first criterion, while Participant 6 just passed the first criterion but did not pass the second.

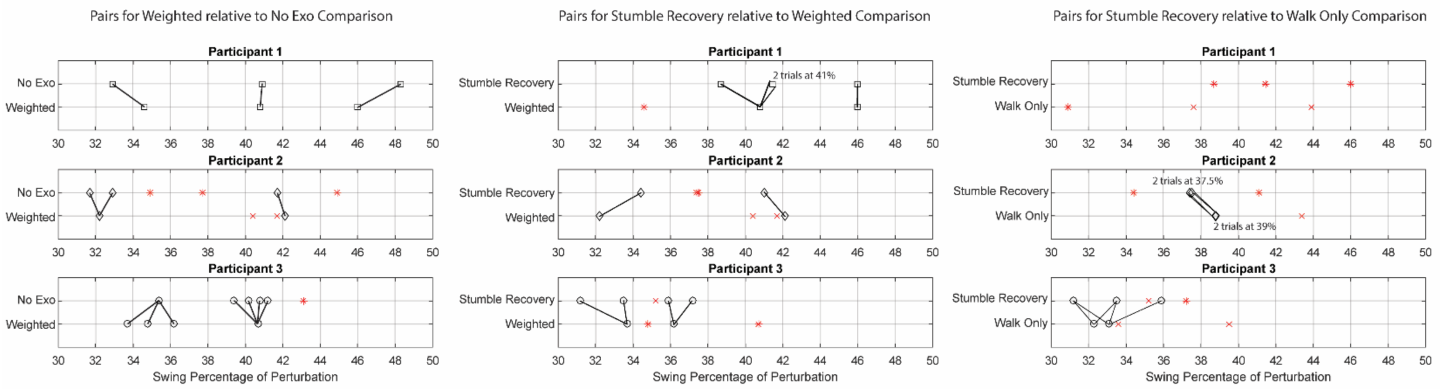

Figure S2. Comparison pairs for Weighted relative to No Exo (left), Stumble Recovery relative to Weighted (middle) and Stumble Recovery relative to Walk Only (right) for each participant. Each stumble that a participant experienced is plotted with swing percentage of perturbation on the x-axis, and Case on the y-axis. Trial pairs (paired according to protocol outlined in Methods) are connected by solid lines. A trial that was excluded due to the block rotating more than 45 degrees is marked with a red 'x'. A trial that was excluded due to timing (i.e., no trial in other Case within 3%) is marked with a red '*'.
